# Supplementary material for: Comprehensive analysis of disulfidptosis-related genes in pulmonary hypertension through machine learning and immune infiltration: Spotlight on USP32 and ZNF655 as key regulators
Source: PLoS One. 2025 Sep 9;20(9):e0330832. doi: 10.1371/journal.pone.0330832 (PMC12419597; doi:10.1371/journal.pone.0330832)
Supplement: Attachment — Submitted filename: Response to Reviewers.docx [file pone.0330832.s004.docx]

Responses to Editor and Reviewer

Dear Academic Editor and Reviewer:

Hello！

We are honored to receive your correspondence. We are grateful for the constructive comments and insightful recommendations provided on our manuscript, titled " Comprehensive analysis of disulfidptosis-related genes in pulmonary hypertension through machine learning and immune infiltration: spotlight on USP32 and ZNF655 as key regulators" We have meticulously revised the original manuscript and addressed each comment in detail. The following section will address each of these issues in turn. We are appreciative of the constructive feedback provided by the editors and reviewers, as it plays a pivotal role in enhancing the quality of our manuscripts. The following section presents a comprehensive, point-by-point response to all comments.

**Academic Editor**

We are very pleased to receive your positive feedback on our manuscript and your reminder for revisions. We have now completed the modifications to the manuscript as requested.

**Comment 1:**

**Please ensure that your manuscript meets PLOS ONE's style requirements, including those for file naming. The PLOS ONE style templates can be found at [https://journals.plos.org/plosone/s/file?id=wjVg/PLOSOne_formatting_sample_main_body.pdf](https://journals.plos.org/plosone/s/file?id=wjVg/PLOSOne_formatting_sample_main_body.pdf) and [https://journals.plos.org/plosone/s/file?id=ba62/PLOSOne_formatting_sample_title_authors_affiliations.pdf](https://journals.plos.org/plosone/s/file?id=ba62/PLOSOne_formatting_sample_title_authors_affiliations.pdf)**

Response 1:

We sincerely appreciate your suggestions. Upon receiving your comments, we promptly revised the manuscript formatting. The current version has now incorporated all these modifications.

**Comment 2:**

**Please note that PLOS ONE has specific guidelines on code sharing for submissions in which author-generated code underpins the findings in the manuscript. In these cases, we expect all author-generated code to be made available without restrictions upon publication of the work. Please review our guidelines at [https://journals.plos.org/plosone/s/materials-and-software-sharing#loc-sharing-code](https://journals.plos.org/plosone/s/materials-and-software-sharing#loc-sharing-code) and ensure that your code is shared in a way that follows best practice and facilitates reproducibility and reuse.**

Response 2:

We sincerely appreciate your suggestions. I visited the URL you provided, but unfortunately, the page displayed an error message indicating it does not exist. As a result, I was unable to proceed with the submission, and I sincerely apologize for this. Could you kindly provide me with detailed submission instructions? I have already uploaded the code to the supplementary materials section.

**Comment 3:**

**Thank you for stating the following financial disclosure:
This study was supported by the Health Development Promotion Project-Anesthesia and Critical Care Research Project (KM-20231120-01), Guangdong Medical Research Fund Project (A2024728、A2024723), the Zhanjiang Science and Technology Research Project in 2022 (No: 2022A01197), and the Science and Technology Development Special Fund Competitive Allocation Project of Zhanjiang City (No: 2021A05086).**

**Please state what role the funders took in the study. If the funders had no role, please state: "The funders had no role in study design, data collection and analysis, decision to publish, or preparation of the manuscript."
If this statement is not correct you must amend it as needed.
Please include this amended Role of Funder statement in your cover letter; we will change the online submission form on your behalf.**

Response 3:

We sincerely appreciate your suggestions.The above funding has contributed to our research, and I have provided a detailed description of its role in the cover letter.

**Comment 4:**

**We note that your Data Availability Statement is currently as follows: The datasets generated and/or analysed during the current study are available from the corresponding author on reasonable request.**

Response 4:

We sincerely appreciate your suggestions.We will submit the original dataset as supplementary material.

**Reviewer #1**

**Comment 1:**

**For a better understanding of the study and datasets, I suggest providing a clear description and discussion of the nature of the datasets used.**

Response 1:

We sincerely appreciate the time you have taken to review our manuscript and are truly honored by your positive feedback. Regarding this section, we have now provided a more detailed description of our methodology in the revised manuscript.

**Comment 2:**

**The findings presented in Figure 1 require further elaboration.**

Response 2:

Thank you for your valuable comments. Regarding the results description for Figure 1, we have provided more detailed revisions in both the main text and the accompanying "Figure" file with enhanced explanations.

**Comment 3:**

**Further elaboration is needed for each panel in Figure 2. What do the modules represent? Do they correspond to genes? How many genes are included in each panel? Why is the grey module considered insignificant?**

Response 3:

Thank you for your valuable comments. In WGCNA (Weighted Gene Co-expression Network Analysis), modules represent clusters of genes with highly correlated expression patterns, where genes serve as the fundamental units of these modules. These modules are constructed through clustering analysis of gene expression data, ensuring that genes within the same module exhibit highly consistent expression trends across diverse samples. Each gene is algorithmically assigned to a specific module or remains unclassified, typically designated as the grey module for genes lacking strong correlation with any defined cluster. The green module contains 1,271 genes, the yellow module contains 950 genes, and the grey module contains 310 genes.

**Comment 4:**

**Figure 3, Is there a correlation between the outcomes of GO enrichment analysis and KEGG enrichment analysis?**

Response 4:

Thank you for your valuable comments. The GO and KEGG enrichment analysis results demonstrate functional correlations in PAH. The enrichment of the TGF-β/SMAD signaling pathway in GO and its association with the EB virus infection pathway in KEGG suggest that viral factors may contribute to PH progression by activating host inflammatory and fibrotic pathways. Simultaneously, the RNA splicing/transport terms in GO and the spliceosome pathway in KEGG collectively indicate that dysregulated RNA metabolism may lead to functional defects in key genes (e.g., BMPR2), driving vascular remodeling. Additionally, the link between phosphatase complexes (GO) and the cytoplasmic DNA-sensing pathway (KEGG) reflects the role of oxidative stress and DNA damage in PH. These findings together reveal a synergistic pathological mechanism in PH characterized by "signaling dysregulation—RNA dysregulation—oxidative stress."

**Comment 5:**

**What criteria led to the selection of the green module over the yellow module for further analysis? Panel G, the list does not seem too extensive. Please provide the gene names instead of numbers.**

Response 5:

Thank you for your valuable comments.Regarding the module selection criteria, we have now provided a more detailed explanation focusing on the results presented in the first section. As for the issue with Figure G, we sincerely apologize for the oversight and have thoroughly revised it in the current version.

**Comment 6:**

**Figure 5, please state the differences between (panel A & panel B) and (panel C & panel D).**

Response 6:

Thank you for your valuable comments. We regret any confusion caused by our initial description. These figures separately display results from the training cohort and validation cohort. For clarity, we have now included comprehensive annotations in the figure legends within the “Figure” file.

**Comment 7:**

**The sentence on page 9/29, starting with “Figure 6 showed that the high...” until the end, is unclear. Please revise it for clarity.**

Response 7:

Thank you for your valuable comments.We sincerely apologize for any inconvenience this may have caused during your review process. This was indeed an issue with our description, which we have now corrected in the manuscript with clear annotations.

**Comment 8:**

**Figure 7, the Fiddle diagram: What is the meaning of “Con” and “Treat”? What kind of differences exist between the cells? Are they related to cell number, gene expression, or other factors?**

Response 8:

Thank you for your valuable comments.“Con” or ‘control’represents the normal group, “Treat” represents the pulmonary hypertension group.This shows the differences in 28 immune cells between the Con (normal) group and the Treat (pulmonary hypertension) group. For example, Natural.Killer.T.cell is mostly highly expressed in the Con group and mostly low - expressed in the Treat group.

**Comment 9:**

**The discussion is lengthy and lacks a well-organized flow. I suggest focusing more on explaining the findings and linking them to the disease.**

Response 9:

Thank you for your valuable comments.Our discussion systematically addresses each experimental finding, with dedicated analysis of all result sections. To enhance reproducibility, we have incorporated comparisons with prior published results to validate the reliability of our current findings.We would be grateful for any additional feedback you may have, and we remain available for further discussion if needed.

**Reviewer #2**

**Comment 1:**

**The figures with higher resolution (text is not completely visible in figures 2, 6, 7, and 8, for example), and more detailed legends are necessary (including experiment descriptions, and how the graphs were generated). This is essential to fully visualize and understand the data, as well as follow the interpretation of the results.**

Response 1:

Thank you for your valuable comments.We sincerely apologize for any inconvenience caused by the image resolution issues during your review. We have now revised all figures using 300dpi TIFF format, which optimizes both clarity and file size. Should you require higher-resolution versions, please don't hesitate to contact me - I would be more than happy to provide whatever file specifications you may need.

**Comment 2:**

**The authors present interesting data produced through a well structured and logical analysis, yet limited by sample size and lack of experimental validation. USP32 and ZNF655 are involved in multiple cellular processes and allow for generating many hypotheses about their role in PAH, therefore, the relevance of their findings could be strengthen by focusing the discussion primarily on how the data connects with what is already well known about the cell and molecular biology of PAH (instead of tackling multiple connections not necessarily as robust).**

Response 2:

Thank you for your valuable comments. Our discussion systematically addresses each experimental finding, with dedicated analysis of all result sections. To enhance reproducibility, we have incorporated comparisons with prior published results to validate the reliability of our current findings.We would be grateful for any additional feedback you may have, and we remain available for further discussion if needed.

**Comment 3:**

**In “Dynamic module identification isolated three significant modules—green, yellow, and gray—with the gray module deemed nonsignificant. Hierarchical clustering further identified the green module as particularly relevant to disease pathology”, can you explain why gray module was deemed nonsignificant? And why is green module is relevant to disease pathology?**

Response 3:

Thank you for your valuable comments.We have revised the relevant descriptions in the manuscript accordingly. However, should you find our explanations still insufficient to address your concerns, please feel free to contact us at any time for more detailed clarification.

**Comment 4:**

**In Figure 2, groups are divided into ‘control’ and ‘treat’. Can you explain what “treat” means?**

Response 4:

Thank you for your valuable comments. “Con” or ‘control’represents the normal group, “Treat” represents the pulmonary hypertension group.This shows the differences in 28 immune cells between the Con (normal) group and the Treat (pulmonary hypertension) group. For example, Natural.Killer.T.cell is mostly highly expressed in the Con group and mostly low - expressed in the Treat group.

**Comment 5:**

**In Figure 4 legend, reference to C is missing.**

Response 5:

Thank you for your valuable comments.We sincerely appreciate your reminder and have now completed the necessary revisions to the figures as suggested.

**Comment 6:**

**In Figure 5, what is the difference between A and B, and C and D?**

Response 6:

Thank you for your valuable comments. We regret any confusion caused by our initial description. These figures separately display results from the training cohort and validation cohort. For clarity, we have now included comprehensive annotations in the figure legends within the “Figure” file.

**Comment 7:**

**Duplicated sentences in discussion: “Furthermore, studies involving bovine subjects have revealed that the expression of olfactory receptors (ORs) is governed by MOR4, a member of the GPCR superfamily. MOR4 is primarily recognized as a binding site for the zinc finger (ZNF) transcription factor gene family, suggesting a link between GPCR activity and ZNF[41]. Furthermore, studies involving bovine subjects have revealed that the expression of olfactory receptors (ORs) is governed by MOR4, a member of the GPCR superfamily. MOR4 is primarily recognized as a binding site for the zinc finger (ZNF) transcription factor gene family, suggesting a link between GPCR activity and ZNF [42].”**

Response 7:

Thank you for your valuable comments.We sincerely apologize for this oversight during manuscript preparation. The corrections have now been properly implemented in the revised version.

The following responses are intended to address the comments that have been submitted. We would like to express our gratitude once again for the time and effort that has been invested in the review of our work.

We extend our best wishes for the success of your future endeavors.

Sincerely,

Dingyu Guo

The Original Submitting Author

E-mail: 1743416277@qq.com
