# Supplementary material for: Comprehensive analysis of disulfidptosis-related genes in pulmonary hypertension through machine learning and immune infiltration: Spotlight on USP32 and ZNF655 as key regulators
Source: PLoS One. 2025 Sep 9;20(9):e0330832. doi: 10.1371/journal.pone.0330832 (PMC12419597; doi:10.1371/journal.pone.0330832)
Supplement: Attachment — Submitted filename: Response_to_Reviewers_auresp_2.docx [file pone.0330832.s005.docx]

Responses to Editor and Reviewer

Dear Academic Editor and Reviewer:

Hello！

We are honored to receive your correspondence. We are grateful for the constructive comments and insightful recommendations provided on our manuscript, titled " Comprehensive analysis of disulfidptosis-related genes in pulmonary hypertension through machine learning and immune infiltration: spotlight on USP32 and ZNF655 as key regulators" We have meticulously revised the original manuscript and addressed each comment in detail. The following section will address each of these issues in turn. We are appreciative of the constructive feedback provided by the editors and reviewers, as it plays a pivotal role in enhancing the quality of our manuscripts. The following section presents a comprehensive, point-by-point response to all comments.

**Academic Editor**

We are very pleased to receive your positive feedback on our manuscript and your reminder for revisions. We have now completed the modifications to the manuscript as requested. In the revised version, we will submit it strictly according to your requirements. Thank you for your continued attention and help to our manuscript.

**Reviewer #1**

Thank you for your suggestions for improvements to our manuscript, and we are honored to have your suggestions along the way.

**Reviewer #2**

**Comment 1:**

**Please clarify whether p-values reported for enrichment analyses, correlation studies, and other statistical tests were adjusted for multiple comparisons (e.g., using FDR or Bonferroni correction).**

Response 1: It's great to receive your suggestions! Thank you for your attention to our manuscript. We clarify that the p-values reported in enrichment analyses, correlation studies, and other statistical tests are not corrected for multiple comparisons, so we did not specifically indicate this in the original text.

**Comment 2:**

**While the construction of ceRNA networks for USP32 and ZNF655 is a potentially valuable addition, the analysis lacks prior introduction or justification for the relevance of miRNA/lncRNA in pulmonary hypertension. Please add a brief explanation and provide the methodological details for this analysis.**

Response 2: It's great to receive your suggestions! Thank you for your attention to our manuscript. Based on your suggestion, we first added a description in the "Introduction" section, followed by a detailed methodological introduction in the "Methods and Results" section, which was also elaborated in the discussion.

**Comment 3:**

**As noted previously, the discussion is overly broad and lacks a cohesive focus. It attempts to associate USP32 and ZNF655 with numerous biological pathways and mechanisms, many of which are tangential or speculative, and dilute the impact of their findings. Consider to streamline the discussion to emphasize a few well-supported, relevant pathways that directly connect their findings to pulmonary hypertension. This would be especially important given that there is no experimental validation of the involvement of USP32 or ZNF655 in pulmonary hypertension in this paper.**

Response 3: It's great to receive your suggestions! Thank you for your attention to our manuscript. Based on your suggestions, we've overhauled and consolidated the original Tall & Thin discussion, highlighting a few of the issues you've highlighted.

**Other comments**

**Comment 1:**

**Figure 4G would be more informative if it contained the gene names instead of numbers.**

Response 1: It's great to receive your suggestions! Thank you for your attention to our manuscript. We have revised the content of Figure 4G.

**Comment 2:**

**Figures 6, 7a and 7c remain difficult to read. Please consider improving its resolution.**

Response2：It's great to receive your suggestions! Thank you for your attention to our manuscript. We've added clarity even further. To ensure clarity, we adjusted the clarity of these figures to 400dpi.

**Comment 3:**

**“Zinc lipoprotein (ZNF) family” appears to be a misstatement. Please revise for accuracy.**

Response3：It's great to receive your suggestions! Thank you for your attention to our manuscript. We're sorry for the inconvenience caused by our mistake, but our team took your suggestion very seriously, and we re-read the literature and found that the original text says "Zinc-finger proteins".The following is the relevant original image.

The following responses are intended to address the comments that have been submitted. We would like to express our gratitude once again for the time and effort that has been invested in the review of our work.

We extend our best wishes for the success of your future endeavors.

Sincerely,

Dingyu Guo

The Original Submitting Author

E-mail: 1743416277@qq.com
